# A Synergistic Antibacterial Study of Copper-Doped Polydopamine on Ti_3_C_2_T_x_ Nanosheets with Enhanced Photothermal and Fenton-like Activities

**DOI:** 10.3390/ma16247583

**Published:** 2023-12-10

**Authors:** Zhuluni Fang, Qingyang Zhou, Wenbo Zhang, Junyi Wang, Yihan Liu, Miao Yu, Yunfeng Qiu, Zhuo Ma, Shaoqin Liu

**Affiliations:** 1 School of Medicine and Health, Harbin Institute of Technology, Harbin 150080, China; 2 School of Life Science and Technology, Harbin Institute of Technology, Harbin 150001, China; zhuoma@hit.edu.cn

**Keywords:** MXene, polydopamine, Fenton−like activity, photothermal effect, antibacterial

## Abstract

In response to the trend of drug−resistant and super bacteria, the existing single antibacterial methods are not sufficient to kill bacteria, and the development of multifunctional antibacterial nanomaterials is urgent. Our study aims to construct copper−doped polydopamine−coated Ti_3_C_2_T_x_ (CuPDA@Ti_3_C_2_T_x_) with an enhanced photothermal property and Fenton−like activity. The nanocomposite hydrogel consisting of CuPDA@Ti_3_C_2_T_x_ and alginate can improve the antioxidant activity of two−dimensional MXene nanosheets by coating them with a thin layer of PDA nanofilm. Meanwhile, Cu ions are adsorbed through the coordination of PDA−rich oxygen−containing functional groups and amino groups. Calcium ions were further used to crosslink sodium alginate to obtain antibacterial hydrogel materials with combined chemotherapy and photothermal therapy properties. The photothermal conversion efficiency of CuPDA@Ti_3_C_2_T_x_ is as high as 57.7% and the antibacterial rate of *Escherichia coli* reaches 96.12%. The photothermal effect leads to oxidative stress in bacteria, increases cell membrane permeability, and a high amount of ROS and copper ions enter the interior of the bacteria, causing protein denaturation and DNA damage, synergistically leading to bacterial death. Our study involves a multifunctional synergistic antibacterial nanodrug platform, which is conducive to the development of high−performance antibacterial agents and provides important research ideas for solving the problem of drug−resistant bacteria.

## 1. Introduction

Antibiotic resistance poses a serious threat to public health, economic growth, and global economic stability [1,2]. In response to the drug resistance of these bacteria, researchers have to synthesize new antibiotics. However, the average cycle time for humans to develop a new antibiotic is about 10 years. In general, most pathogenic bacteria reproduce every 20 min and may develop resistance within 2 years, leading to a dead cycle in new drug development [3,4]. Therefore, there is an urgent need for new antibacterial agents or treatment strategies to combat highly resistant bacteria. Previous work has found that nanomaterials have higher antibacterial properties due to their quantum confinement effect, small size effect, high surface area to volume ratio, and highly exposed surface atoms [5,6,7]. At present, metal−based [8,9,10,11,12], carbon−based [13,14], and polymer−based antibacterial nanomaterials [15], have displayed promising antibacterial properties.

On the basis of the photoconversion function of nanomaterials, local high heat can be generated upon infrared−light irradiation and can effectively kill bacteria [16,17,18]. Thus, the photothermal therapy (PTT) antibacterial strategy was developed and has attracted tremendous attention in the antibacterial field [18,19]. This treatment mechanism does not consider bacterial gene mutations and can avoid causing bacterial resistance. Compared to traditional antibiotic treatment strategies, the bacterial resistance caused by PTT is almost negligible [20]. The main obstacle of PTT is the development of nanomaterials with a high light−conversion efficiency [21]. MXene is a new multifunctional, two−dimensional nanomaterial, mainly used by the Gogotsi research group, and generally includes transition metal carbides, nitrides, and carbonitrides [22,23]. Recently, some studies have revealed the physical membrane destruction effect of MXene, which may exhibit a unique biological activity to kill pathogens [24]. On the one hand, the interaction between MXene (the sharp edges of nanosheets that act as “nanoknives”) and cell membranes may damage the integrity of bacterial membranes during physical contact, ultimately leading to cell death [25,26]. On the other hand, MXenes with zero band gaps have an excellent photothermal conversion efficiency, enabling them to act as photothermal agents for antibacterial applications [27,28,29]. Recently, Sun et al. constructed a photothermal nanoantibacterial material based on MXene to effectively kill methicillin−resistant *Staphylococcus aureus* (MRSA) by regulating the activity of lysozyme [30]. Wan et al. developed a sensor based on an antibacterial MXene hydrogel, which could effectively accelerate wound healing and achieve a human–computer interaction [31]. Wang et al. created a series of chitin/MXene composite sponges to prevent considerable blood loss and promote the healing process of bacteria−infected wounds [32].

However, Ti_3_C_2_T_x_ in the MXene family is unstable and is easily oxidized into TiO_2_ when stored in air in an ambient condition, especially at a relatively higher temperature, thus losing its photothermal function. Due to its high negative surface charge, it is usually difficult to electrostatically bind Ti_3_C_2_T_x_ onto anionic bacterial membranes to achieve effective physical destruction. Therefore, strategies, such as structural modification and drug compatibility, may be needed to simultaneously enhance the antibacterial activity and selectivity of Ti_3_C_2_T_x_. Combination therapy is a widely adopted concept in bacterial therapy, which improves treatment efficiency by combining two different strategies compared to using a specific therapy alone. Therefore, the multifunctional nanoplatform based on photothermal, photodynamic, chemical antibacterial activities needs additional investigations for finally developing highly efficient broad−spectrum antibacterial nanoagents [33,34,35].

This paper presents a Ca^2+^ crosslinking alginate hydrogel film consisting of CuPDA−coated Ti_3_C_2_T_x_, which possesses photothermal and chemical antibacterial activities. Ti_3_C_2_T_x_ with a zero band gap shows an excellent photothermal conversion efficiency. PDA belongs to the melanin class and is selected due to its film−forming coordination ability and biocompatible properties. Cu^+^ presents Fenton−like activity to produce •OH according to Equation (1): ${Cu}^{+}{+H_{2}O}_{2}+H^{+}\to {Cu}^{2+}+\cdot OH+H_{2}O$ [36]. Alginate is an excellent hydrogel agent and its mechanical strength can be augmented by Ca^2+^ crosslinking. Thus, Cu ion−coordinated PDA is used to coat Ti_3_C_2_T_x_ to prevent its surface oxidation and enhance its biocompatibility and photothermal activity [37]. And the alginate−based hydrogel film containing CuPDA−coated Ti_3_C_2_T_x_ might possess synergistic photothermal and Fenton−like properties, and displays an excellent sterilization function. Therefore, this project is expected to provide important material design ideas and antibacterial mechanism research foundations for the development of high−performance synergistic antibacterial nanomaterials.

## 2. Materials and Methods

### 2.1. Materials

Ti_3_AlC_2_, dopamine hydrochloride (98.0%), dimethyl sulfoxide (99.7%), CuCl_2_, HCl, and LiF were purchased from Aladdin Co., Ltd. (Shanghai, China). Tris−base (C_4_H_11_NO_3_, 99.9%) was bought from Macklin Biochemical Co., Ltd. (Shanghai, China). *E. coli* and *S. aureus* were bought from Biobw Biotechnology Co., Ltd. (Beijing, China). PBS, nutrient broth (NB), and nutrient agar (NA) were acquired from Bio−Channel Biotechnology Co., Ltd. (Nanjing, Jiangsu, China).

### 2.2. Characterization

The nanosheet structure was determined using scanning electron microscopy (SEM) Quanta FEG (Thermo Fisher Scientific Inc., Waltham, MA, USA) and transmission electron microscopy (TEM) Tecnai G2 (Thermo Fisher Scientific Inc., Waltham, MA, USA), including the characterization of the nanomorphology and size of Ti_3_C_2_T_x_ and its composites. The phase of the sample was measured using X−ray diffraction (XRD) (Rigaku D/Max 2500 PC, Rigaku Corporation, Tokyo, Japan). Renishaw Invia Raman microscope (Beijing, China) was used to determine the functional groups of the sample, and X−ray photoelectron spectroscopy (XPS) using K−Alpha anode (Thermo Scientific, Waltham, MA, USA) was used to determine the surface elemental composition and valence of the sample. We measured the absorbance and photothermal conversion efficiencies of the sample using a UV−Vis−NIR spectrophotometer (Agilent Technologies, Santa Clara, CA, USA) and an infrared thermal imager (FLIR E8, Oregon, Portland, OR, USA). F−320 fluorescence spectrophotometer (Tianjin, China) was used to determine •OH radicals using a terephthalic acid (TA) probe.

### 2.3. Synthesis of Ti_3_C_2_T_x_ Nanomaterials

In order to prepare mud−like Ti_3_C_2_T_x_, this experiment used a mixed solution of concentrated hydrochloric acid and lithium fluoride to soak the Ti_3_AlC_2_, and the Al element was etched by in situ−generated HF. The mixture was stirred continuously at 35 °C for 24 h, with N_2_ bubbling to prevent oxidation. After the completion of the reaction, we washed the precipitate with a high quality of deionized water and measured the pH value until a gray−black turbid solution was obtained. The mixture was centrifuged at 3500 rpm for 5 min and treated with nitrogen gas. The upper layer of the turbid liquid was collected, washed multiple times, and vacuum−dried.

### 2.4. Synthesis of CuPDA@Ti_3_C_2_T_x_

The Ti_3_C_2_T_x_ dispersion was prepared and coated with PDA on the surface using the alkaline polymerization method. The mixture was adjusted to a pH of 8.5 using a Tris. A dopamine monomer and copper chloride were added, and then static polymerization was maintained for 24 h in the presence of oxygen. The CuPDA@Ti_3_C_2_T_x_ powder was centrifuged and vacuum−dried for use.

### 2.5. Preparation of CuPDA@Ti_3_C_2_T_x_ Alginate Hydrogel

At 25 °C, 50 mg of sodium alginate was dispersed under ultrasound in the CuPDA@Ti_3_C_2_T_x_ solution (0.4 μg/mL). A total of 1 mL of the CaCl_2_ solution (50 μg/mL) was added to the suspension to start the crosslinking reaction. The CuPDA@Ti_3_C_2_T_x_ hydrogel was collected and purified with water three times, until no residue was detected in the spectral test.

### 2.6. Photothermal Property Calculation

The photothermal performance of the nanocomposites was measured under 808 nm near−infrared (NIR) laser irradiation [38]. The concentrations of various composite suspensions ranged from 100 to 500 μg/mL. Then, different concentrations of nanocomposite suspensions were irradiated with a near−infrared laser (808 nm, 1.0 W/cm^2^), and temperature−changed and thermal images were recorded every 30s using thermal imaging technology for 10 min. The photothermal conversion efficiency was calculated according to the following formula:

(1) $$\theta =\frac{T-T_{surr}}{T_{max}-T_{surr}}$$

(2) $$\tau_{s}=\frac{t}{-\ln\theta }$$

(3) $$hS=\frac{Cm}{\tau_{s}}$$

(4) $$Q_{dis}=hS(T_{max}-T_{surr})$$

(5) $$\eta_{1}=\frac{hS\left(T_{max}-T_{surr}\right)-Q_{dis}}{I(1-10^{-A_{808}})}$$

where *η_1_* is the material’s photothermal conversion efficiency value, *T_max_* is the maximum temperature of the photothermal material dispersion or deionized water, *T_surr_* is the ambient temperature, *A*_808_ is the absorbance of the dispersion at 808 nm, *Q_dis_* is the heat emitted by the environment, *C* is the specific heat capacity of water, T is the real−time temperature of the photothermal material dispersion or deionized water during the temperature measurement, *t* is the time, *τ_S_* is the slope of the cooling curve fitting line for the water dispersion or deionized water of the photothermal material, and *I* is the power density of the laser used.

### 2.7. Antibacterial Measurement

The colony counting method was used to determine the number of colony forming units (CFUs) for CuPDA@Ti_3_C_2_T_x_ under near−infrared irradiation. *E. coli* was incubated in fresh MHB in a shaking incubator at 37 °C. Logarithmic metaphase cells were diluted to 2 × 10^5^ CFU/mL, and 500 μL of PDA@Ti_3_C_2_T_x_ nanocomposite was added. The mixture was shaken continuously at 37 °C for 1 h. Subsequently, cells from the Ti_3_C_2_T_x_ + NIR, PDA@Ti_3_C_2_T_x_ + NIR, and CuPDA@Ti_3_C_2_T_x_ + NIR groups were monitored using an infrared thermal imager. After another 5 h of cultivation, each group of bacterial suspension was continuously diluted 10 times and placed on an MHA plate, which was then incubated overnight at 37 °C. The bacterial colonies were counted and imaged on the board. All experiments were repeated three times. The staining method for live and dead bacteria was performed using Syto−9 to generate green fluorescence, which could characterize live bacteria, and propidium iodide (PI) could emit red fluorescence to characterize dead bacteria.

## 3. Results

**Figure 1:**
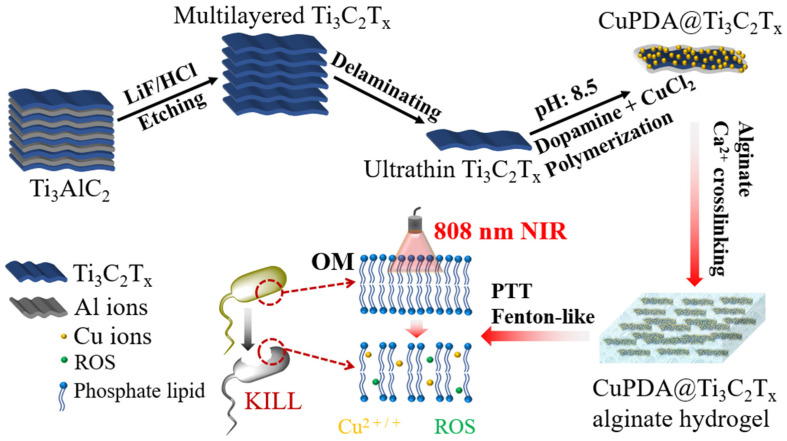
Illustration of preparation procedure and antibacterial function of CuPDA@Ti_3_C_2_T_x_ nanocomposite hydrogel materials.

As shown in Figure 1, the design concept of this study was to use LiF and concentrated HCl routes to etch Ti_3_AlC_2_, create Ti_3_C_2_T_x_ nanosheets, and coat them with Cu−coordinated polydopamine to improve their water–oxygen stability while reducing cell toxicity [39,40]. The abundant oxygen−containing functional groups and amino coordination of polydopamine were utilized to adsorb Cu^2+^. Finally, Ca^2+^ ions crosslinked with sodium alginate hydrogel were used to prepare a composite antibacterial dressing, and the photothermal effect was used to improve the permeability of the bacterial membrane, promote the penetration capabilities of ROS and Cu ions, and synergistically improve its antibacterial performance.

### 3.1. Synthesis and Characterization of Nanocomposites

**Figure 2:**
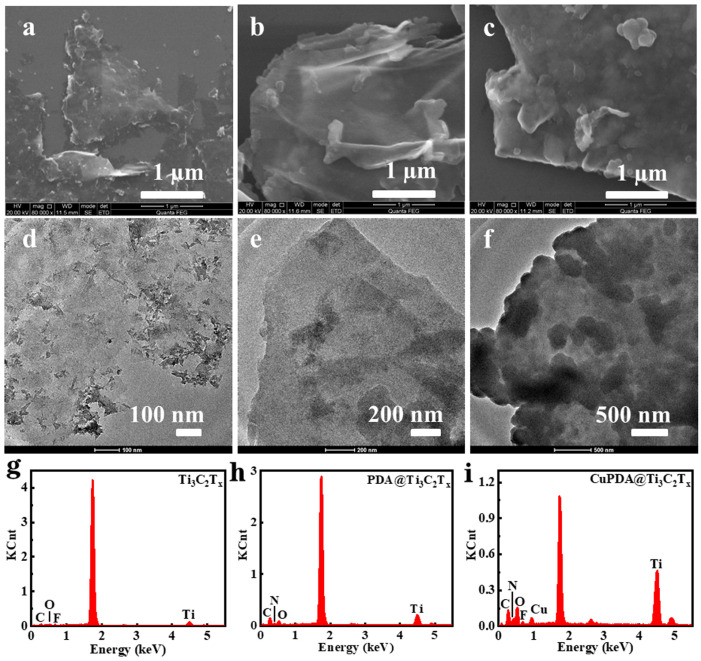
SEM and TEM images of (**a**,**d**) Ti_3_C_2_T_x_, (**b**,**e**) PDA@Ti_3_C_2_T_x_, and (**c**,**f**) CuPDA@Ti_3_C_2_T_x_. Corresponding EDS spectra for (**g**) Ti_3_C_2_T_x_, (**h**) PDA@Ti_3_C_2_T_x_ and (**i**) CuPDA@Ti_3_C_2_T_x_.

From the SEM images in Figure 2a, it can be seen that Ti_3_C_2_T_x_ exhibits a layered structure. In general, the sample exfoliated with HF exhibited the morphology of an accordion. However, the mixed exfoliation method of LiF and concentrated HCl could obtain the MXene mud, which was more suitable as a two−dimensional support for further loading other antibacterial agents. Figure 2b,c are the SEM images of PDA@Ti_3_C_2_T_x_ and CuPDA@Ti_3_C_2_T_x_, which show a rough surface and increased thickness, indicating that the Ti_3_C_2_T_x_ material successfully coated the PDA polymer on its surface. As shown in Figure 2d,f, compared with the TEM image of Ti_3_C_2_T_x_, there are some thin films and granular substances on the surface of the PDA@Ti_3_C_2_T_x_ and CuPDA@Ti_3_C_2_T_x_ materials, which may be caused by PDA or CuPDA coating.

As shown in Figure 2g, there are characteristic peaks for Ti, C, O, and F in the energy dispersive spectrometer (EDS) spectrum of Ti_3_C_2_T_x_, with element contents of 3.25%, 20.1%, 4.8%, and 1.3%, respectively. In the case of PDA@Ti_3_C_2_T_x_ in Figure 2h, there are characteristic peaks for Ti, C, O, and N in the EDS spectrum; the corresponding element contents are 4.2%, 38.3%, 9.0%, and 7.8%. After introducing Cu ions, CuPDA@Ti_3_C_2_T_x_ showed characteristic peaks of Ti, C, O, N, F, and Cu in the EDS spectrum, and the Cu content was ca. 1.5%. The N element came from PDA and Cu was produced due to the coordination between PDA and Cu, indicating that the surface of Ti_3_C_2_T_x_ was successfully coated by the Cu−coordinated PDA.

**Figure 3:**
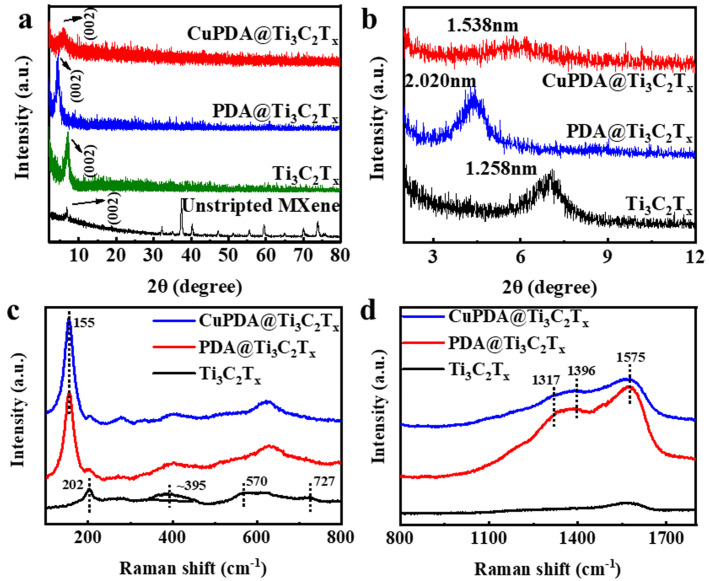
(**a**) XRD patterns for unstripped Ti_3_AlC_2_ and exfoliated Ti_3_C_2_T_x_, PDA@Ti_3_C_2_T_x_, and CuPDA@Ti_3_C_2_T_x_. (**b**) Enlarged XRD patterns in small angles for the calculation of the interlayer distance in corresponding materials. (**c**,**d**) Raman spectra of exfoliated Ti_3_C_2_T_x_, PDA@Ti_3_C_2_T_x_, and CuPDA@Ti_3_C_2_T_x_ in different wavenumber regions.

X−ray diffraction (XRD) further indicates the successful synthesis of exfoliated Ti_3_C_2_T_x_, PDA@Ti_3_C_2_T_x_, and CuPDA@Ti_3_C_2_T_x_ in Figure 3a,b, compared with unstripped Ti_3_AlC_2_, and the main characteristic peak appears at 2θ = 6° for the Ti_3_C_2_T_x_ nanosheets [41]. According to the Bragg equation, the interlayer spacing increased to 1.258 nm, confirming the successful delamination of Ti_3_AlC_2_ and the formation of the Ti_3_C_2_T_x_ nanosheets. The characteristic peak for PDA@Ti_3_C_2_T_x_ shifted to a low angle and the interlayer spacing increased to 2.020 nm, confirming the successful encapsulation of PDA between the adjacent Ti_3_C_2_T_x_ nanosheets. The addition of Cu ions reduced the interlayer distance to 1.538 nm, attributed to the electrostatic repulsion of positively charged Cu ions, which reduced the interlayer distance.

The Raman spectra further confirmed the composition of the antibacterial material. The peaks of 202, 395, and 570 cm^−1^ are attributed to −O, −F, and −OH, respectively, on the surface of Ti_3_C_2_T_x_ in Figure 3c. As seen in Figure 3d, three characteristic peaks can be observed for PDA@Ti_3_C_2_T_x_ and CuPDA@Ti_3_C_2_T_x_, appearing at 1317, 1396, and 1575 cm^−1^, which belong to the −CH_2_NH_2_, C−C/C−N, and C−N indole groups of PDA, respectively [42]. Thus, the Raman results show the successful coating of PDA on the surface of Ti_3_C_2_T_x_.

**Figure 4:**
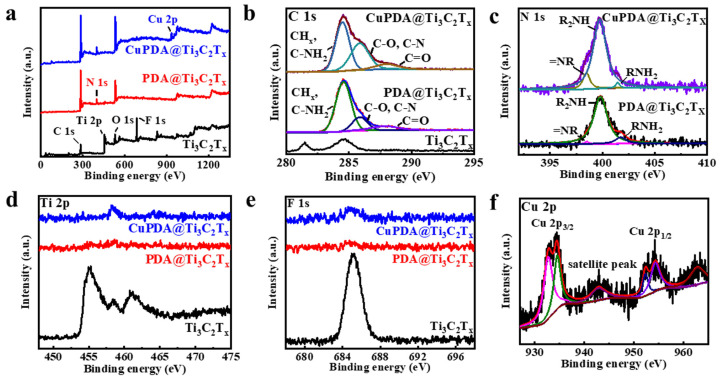
(**a**) Full XPS spectra for Ti_3_C_2_T_x_ (black curve), PDA@Ti_3_C_2_T_x_ (red curve), and CuPDA@Ti_3_C_2_T_x_ (blue curve), and fine deconvoluted XPS spectra for (**b**) C 1s, (**c**) N 1s, (**d**) Ti 2p, (**e**) F 1s, and (**f**) Cu 2p.

X−ray photoelectron spectroscopy (XPS) is a non−destructive measurement method that can analyze the elemental composition and valence state of elements. Figure 4a shows the full XPS spectra of each sample, and the characteristic peaks of C 1s, Ti 2p, O 1s, and F 1s can be observed, located at 284.6, 455.1, 529.6, and 685.1 eV, respectively, indicating that the Ti_3_C_2_T_x_ material has been successfully synthesized. It can be seen that the Al signal is not observable, further indicating that Al has been effectively etched from Ti_3_AlC_2_. In addition, the XPS full spectrum indicates that PDA@Ti_3_C_2_T_x_ contains 6.46% N, confirming a successful PDA−coating process. The N 1s spectrum (Figure 4b) indicates an increase in the characteristic peak area of =NR, possibly due to the N coordination effect between Cu and PDA [43]. As shown in Figure 4d,e, the Ti 2p and F 1s peaks significantly weaken, attributed to the XPS testing depth of only 5–10 nm, which cannot penetrate the PDA coating. This indicates that Ti_3_C_2_T_x_ nanosheets are almost completely encapsulated by PDA, which is beneficial for improving their antioxidant property. As seen in Figure 4f, the Cu ion 2p3/2 and 2p1/2 spin−orbit coupling splitting peaks appear at binding energies of 932.8 and 952.3 eV [44], indicating the presence of Cu^+^, while the splitting peaks at 934.5 and 954.3 eV indicate the presence of Cu^2+^. It is assumed that the Fenton−like activity of Cu^+^ will improve the antibacterial ability to some extent [1].

**Figure 5:**
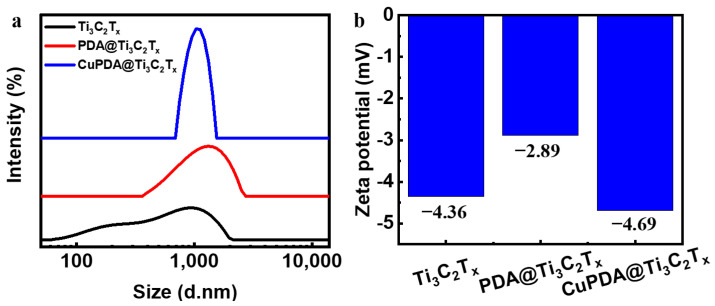
(**a**) Particle size distribution diagram and (**b**) zeta−potential diagram for Ti_3_C_2_T_x_, PDA@Ti_3_C_2_T_x_, and CuPDA@Ti_3_C_2_T_x_.

The dynamic light−scattering (DLS) measurement in Figure 5a shows that the average size of Ti_3_C_2_T_x_ nanosheets is about 1000 nm, and the hydration particle size increases to about 1200 nm after PDA coating. The zeta−potential results of the three materials in Figure 5b show that there is only a low level of negative charge on the surface.

### 3.2. Photothermal Properties

**Figure 6:**
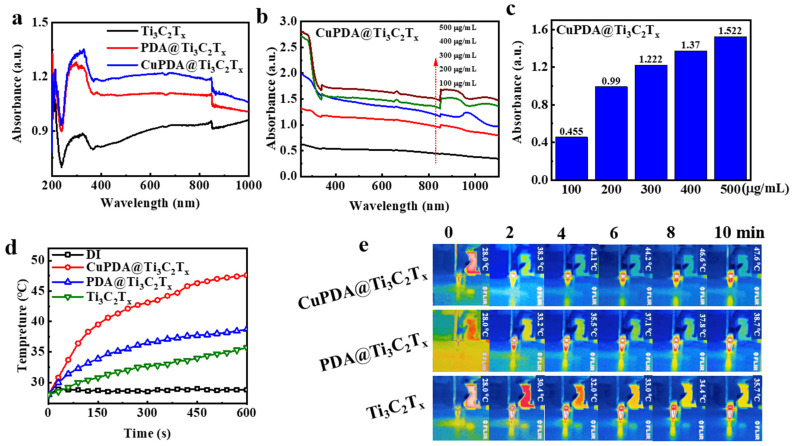
(**a**) UV−Vis−NIR diffuse reflection spectra (DRS) of Ti_3_C_2_T_x_ (black curve), PDA@Ti_3_C_2_T_x_ (red curve), and CuPDA@Ti_3_C_2_T_x_ (blue curve). (**b**) UV−Vis−NIR absorbance spectra of CuPDA@Ti_3_C_2_T_x_ at various concentrations, arrow indicated the concentrations increase from 100 to 500 µg/mL, and (**c**) corresponding absorbance dependence histogram at 808 nm. (**d**) Solution temperature−rise curves for pure distilled water (DI), Ti_3_C_2_T_x_, PDA@Ti_3_C_2_T_x_, and CuPDA@Ti_3_C_2_T_x_ water dispersion at a concentration of 500 µg/mL. (**e**) Thermal imaging maps for Ti_3_C_2_T_x_, PDA@Ti_3_C_2_T_x_, and CuPDA@Ti_3_C_2_T_x_ dispersed solutions.

To verify the near−infrared (NIR) absorption performance of the material, we tested the DRS spectra of various photothermal materials. As shown in Figure 6a, the absorbance intensity of Ti_3_C_2_T_x_ at 808nm is approximately 0.934. After coating the PDA, its absorbance increased to 1.099. When further coordinated with copper ions, CuPDA@Ti_3_C_2_T_x_ had the highest absorbance intensity, reaching 1.19, which was conducive to improving the photothermal conversion performance. Figure 6b compares the UV−Vis−NIR absorbance spectra for CuPDA@Ti_3_C_2_T_x_ under different concentration conditions. When the dispersion concentration increased from 100 to 500 µg/mL, the higher the concentration, the greater the absorption intensity. As shown in Figure 6c, the absorbance of the composite suspension exhibits a concentration−dependent relationship at a wavelength of 808 nm.

The potential of composite materials in photothermal antibacterial applications was verified by measuring the temperature evolution profile of laser irradiation. As shown in Figure 6d, the temperature of suspensions of various materials at 500 µg/mL rapidly increases under an 808 nm laser irradiation of 1 W/cm^2^, and the surface temperature of Ti_3_C_2_T_x_ increases to 35.7 ° C after 10 min of irradiation. However, the surface temperature of CuPDA@Ti_3_C_2_T_x_ can be raised to 47.6 ° C. In contrast, there is no significant temperature change in the temperature of water, indicating that CuPDA@Ti_3_C_2_T_x_ can efficiently and rapidly convert NIR light into thermal energy. Correspondingly, we recorded the thermal imaging maps of the dispersed solutions in the EP tubes during 10 min of 808 nm NIR irradiation (Figure 6e). It can be seen that the CuPDA@Ti_3_C_2_T_x_ dispersion exhibits a uniform higher−temperature distribution than Ti_3_C_2_T_x_ and PDA@Ti_3_C_2_T_x_, indicating its better photothermal ability.

**Figure 7:**
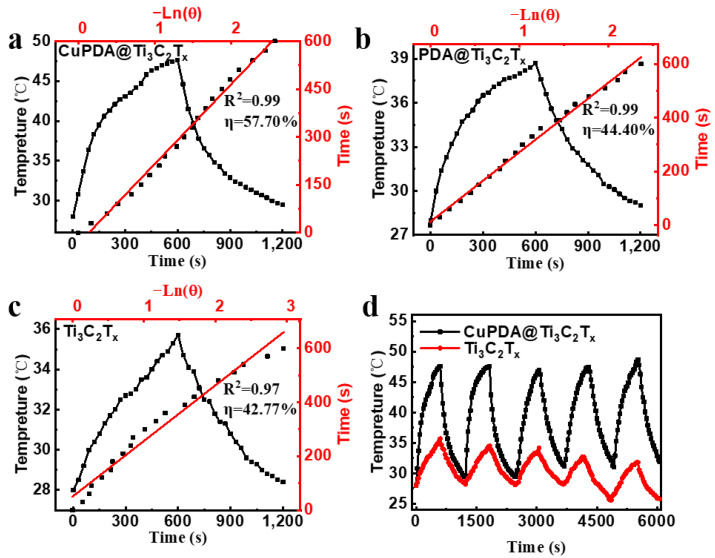
Photothermal conversion efficiencies of (**a**) CuPDA@Ti_3_C_2_T_x_, (**b**) PDA@Ti_3_C_2_T_x_, and (**c**) Ti_3_C_2_T_x_, red line is the linear fitting curve. (**d**) Stability curves of photothermal heating–cooling for CuPDA@Ti_3_C_2_T_x_ (black curve) and Ti_3_C_2_T_x_ (red curve).

According to Equations (1)–(5), the photothermal conversion efficiencies of Ti_3_C_2_T_x_ and PDA@Ti_3_C_2_T_x_ are 42.77% (Figure 7a) and 44.4% (Figure 7b), while the photothermal conversion efficiency of CuPDA@Ti_3_C_2_T_x_ significantly improves, reaching 57.7% (Figure 7c), which is higher than most reported NIR photothermal conversion materials, such as Ti_3_C_2_T_x_ (30.6%) [45], MXene@EGCG (29.2%) [46], and Mo_4_VC_4_ (45.5%) [47]. To verify the photothermal stability of the material, the heating and cooling curves of the photothermal materials were tested (Figure 7d). The temperature of the CuPDA@Ti_3_C_2_T_x_ suspension remained stable at 47.6 °C after five cycles, while the temperature of the uncoated material decreased by 3.9 °C, indicating the potential of CuPDA@Ti_3_C_2_T_x_ as a durable photothermal agent for PTT.

**Figure 8:**
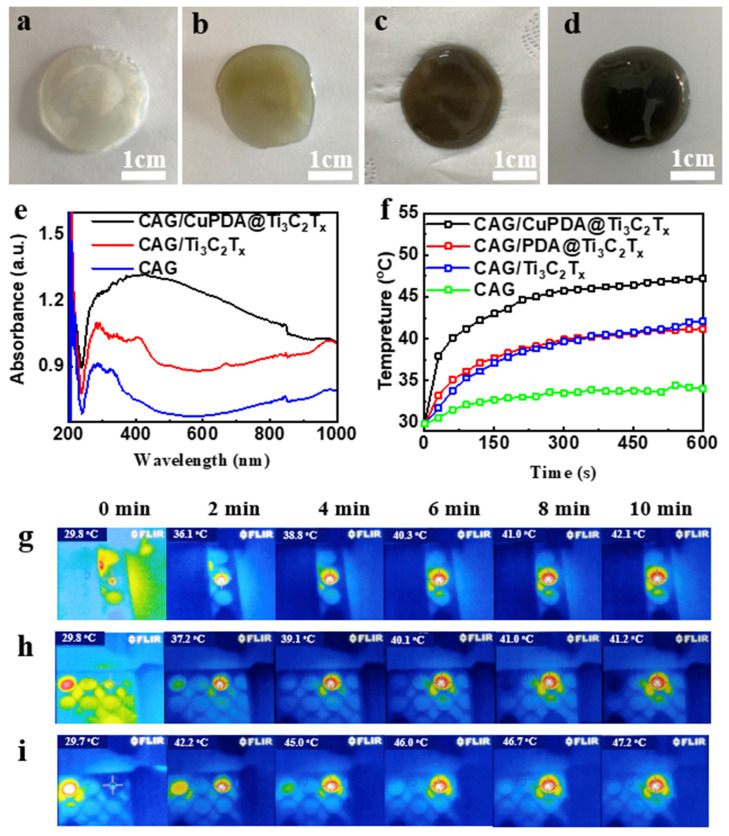
Digital pictures of (**a**) CAG, (**b**) CAG/Ti_3_C_2_T_x_, (**c**) CAG/PDA@Ti_3_C_2_T_x_, and (**d**) CAG/CuPDA@Ti_3_C_2_T_x_ hydrogels. (**e**) Corresponding DRS and (**f**) surface temperature−rise curves. Thermal imaging maps for (**g**) CAG/Ti_3_C_2_T_x_, (**h**) CAG/PDA@Ti_3_C_2_T_x_, and (**i**) CAG/CuPDA@Ti_3_C_2_T_x_ hydrogels. Blue represents low temperature.

At 25 °C, 50 mg of sodium alginate was dispersed into Ti_3_C_2_T_x_, PDA@ Ti_3_C_2_T_x_, and CuPDA@ Ti_3_C_2_T_x_ solutions (500 μg/mL) under ultrasonication conditions. A total of 1 mL of CaCl_2_ solution (50 μg/mL) was added to the suspension to start the crosslinking reaction, as shown in Figure 8a–d. After 2 h, a stable gel film was formed and the color changed from colorless to light brown and finally to black. The total concentration of antibacterial nanomaterials in the gel was about 10 µg/mg; such a relatively low nanomaterial content might decrease the cytotoxicity. As shown in Figure 8e, the hydrogel has obvious absorption properties at 808 nm in the near−infrared region, and the CAG/CuPDA@Ti_3_C_2_T_x_ hydrogel has the strongest absorbance at 808 nm. The surface temperature of the blank CAG hydrogel increased to 34 °C after 10 min of irradiation with a 1 W/cm^2^ 808 nm laser (Figure 8f). However, the surface temperature of the Ti_3_C_2_T_x_ hydrogel increased to 42.1 °C after 10 min of irradiation due to the absorption of Ti_3_C_2_T_x_ in the NIR region. After testing the temperature of the CAG/CuPDA@Ti_3_C_2_T_x_ hydrogel further, the surface temperature increased to 47.2 °C, which indicated that CAG/CuPDA@Ti_3_C_2_T_x_ could efficiently and rapidly convert near−infrared light into thermal energy, and the effect was better than that of CAG@Ti_3_C_2_T_x_. At the same time, we also recorded the thermal imaging maps of CAG/Ti_3_C_2_T_x_ (Figure 8g), CAG/PDA@Ti_3_C_2_T_x_ (Figure 8h), and CAG/CuPDA@Ti_3_C_2_T_x_ (Figure 8i) hydrogels irradiated by an 808 nm NIR value for 10 min. The CAG/CuPDA@Ti_3_C_2_T_x_ hydrogel film showed the highest surface temperature and exhibited a uniform temperature distribution, consistent with the thermal imaging maps for the EP tubes.

### 3.3. Antibacterial Performance and Mechanism

**Figure 9:**
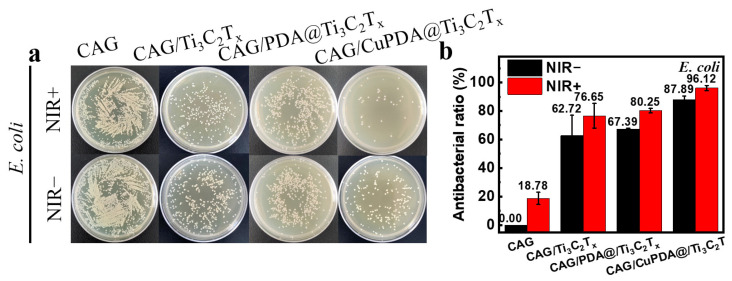
(**a**) Plate—counting results after the co—cultivation of *E. coli* and (**b**) corresponding antibacterial rates for CAG, CAG/Ti_3_C_2_T_x_, CAG/PDA@Ti_3_C_2_T_x_, and CAG/CuPDA@Ti_3_C_2_T_x_ hydrogels.

In this experiment, *E. coli* was selected to evaluate the antibacterial activity of a nanocomposite antibacterial hydrogel. As shown in Figure 9a, after co−culturing with bacteria at 37 °C for 6 h, the number of colonies in CAG, CAG/Ti_3_C_2_T_x_, CAG/PDA@Ti_3_C_2_T_x_, and CAG/CuPDA@Ti_3_C_2_T_x_ decreased sequentially, indicating that the CAG/CuPDA@Ti_3_C_2_T_x_ hydrogel presented stronger antibacterial properties. As expected, the number of bacterial colonies in CAG did not change significantly after light treatment. In order to compare the antibacterial properties of various materials, the antibacterial rate was calculated. As shown in Figure 9b, under near−infrared−light conditions, the antibacterial rate of the CAG hydrogel is only 18.78%. When mixed with the NIR−light−absorbing material, Ti_3_C_2_T_x_, its antibacterial rate increased to 76.65%. Furthermore, Ti_3_C_2_T_x_ coated with PDA was mixed into the gel. Based on the spontaneous H_2_O_2_ generation of PDA and the photothermal function of the composite gel, its antibacterial rate increased to 80.25% [48]. It is worth mentioning that, with the introduction of Cu ions, the antibacterial efficiency of the CAG/CuPDA@Ti_3_C_2_T_x_ composite hydrogel group against *E. coli* reached 96.12%, indicating that our hydrogel presented excellent antibacterial properties due to the Fenton−like reaction and photothermal effect of copper ions.

## 4. Discussion

**Figure 10:**
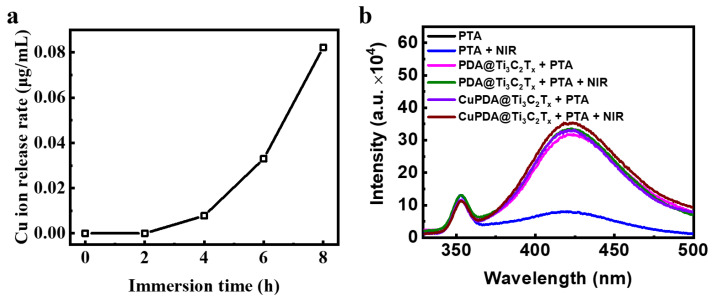
(**a**) Cu ion release curve for CAG@CuPDA@Ti_3_C_2_T_x_. (**b**) Fluorescence spectra in the presence of PTA with and without NIR irradiation.

To explore the sterilization mechanism of hydrogels, the release of Cu ions was tested (Figure 10a). The results show that, after soaking them in PBS for 6 h, the concentration of Cu ions in the prepared solution reached 0.033 μg/mL, indicating that the composite hydrogel could release Cu ions. According to the XPS results, there are Cu^+^ ions that present Fenton−like activity to produce •OH. To verify the species of ROS, sodium terephthalate (PTA) was used as a fluorescence probe to detect the •OH in the solution [49]. PTA is a fluorescent reagent that undergoes a fluorescence enhancement reaction in the presence of •OH, and a stronger fluorescence intensity indicates the presence of more •OH. •OH radicals undergo an additional reaction with PTA, forming a stable form of 2−hydroxyterephthalic acid within the molecule. This stable form is more prone to fluorescence resonance energy transfer than terephthalic acid itself, thereby enhancing the fluorescence signal intensity. As shown in Figure 10b, under both light and dark conditions, the fluorescence intensity of the PTA solution at 425 nm was low and almost unchanged. After mixing CAG/PDA@Ti_3_C_2_T_x_, the fluorescence intensity significantly increased, indicating that the nanomaterial could produce more •OH. According to the electrochemical potential of PDA, its oxidation potential to quinone is ca. 0.2 V, while the electrochemical potential of O_2_/H_2_O_2_ is ca. 0.5 V [50]. Therefore, in thermodynamics, PDA can spontaneously oxidize to generate H_2_O_2_. As expected, the CAG@CuPDA@Ti_3_C_2_T_x_ solution showed the highest fluorescence intensity, indicating that more •OH radicals were generated, which was related to the Fenton−like effect of Cu^+^ [51].

**Figure 11:**
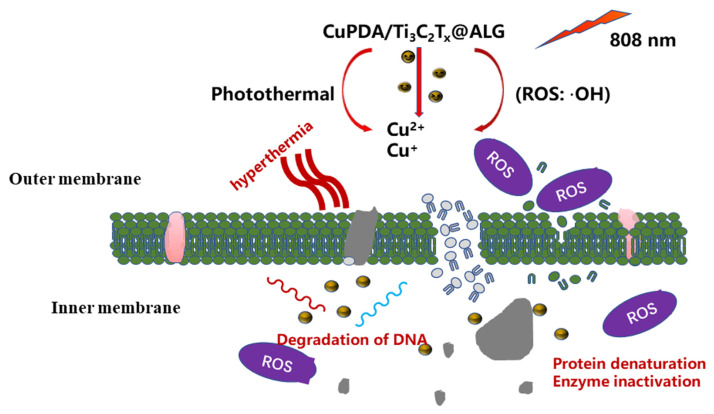
Proposed antibacterial mechanism of the CAG@CuPDA@Ti_3_C_2_T_x_ upon irradiation.

In brief, the combination of copper ion chemical antibacterial and PTT antibacterial therapies can effectively produce antibacterial effects through NIR irradiation [52]. As shown in Figure 11, when exposed to 808 nm NIR irradiation, CAG@CuPDA@Ti_3_C_2_T_x_ can convert light energy into heat energy, resulting in a thermal effect that alters the structure of bacterial cell membranes. This alteration leads to changes in bacterial cell membrane permeability, and in extreme cases, high heat levels can directly cause a bacterial membrane rupture [53,54]. At the same time, a certain number of H_2_O_2_ molecules are produced due to the spontaneous self−oxidation of PDA in the presence of O_2_. Then, the Cu^+^ Fenton−like reaction, as described above, is used to catalyze H_2_O_2_ into more oxidative form of •OH. Due to the increase in the outer−membrane permeability caused by photothermal treatment, Cu^2+^ and Cu^+^ ions, as well as •OH, enter the interior of the cells, damaging the proteins, DNA, and other structures. Therefore, this damage affects the normal physiological activities of bacteria and leads to bacterial lysis and death.

## 5. Conclusions

In conclusion, we reported the Fenton−like and photothermal effects of the CAG hydrogel, and also studied the synergistic antibacterial effect of its chemical kinetics and photothermal effect. After coating the surface of Ti_3_C_2_T_x_ with PDA, its antioxidant activity can be significantly improved, which is beneficial for obtaining high photothermal stability. At the same time, the spontaneous oxidation reaction of the catechol structure in PDA can be utilized to generate H_2_O_2_. Therefore, PDA can generate Cu^+^ ions by coordinating the Cu ions with abundant chemical functional groups, and its Fenton−like reaction can be used to reduce H_2_O_2_ to produce •OH. Compared with pure Ti_3_C_2_T_x_, our antibacterial material exhibited greater photothermal stability in five heating−cooling cycle experiments. This photothermal effect can improve the permeability of the bacterial outer membrane, and the Cu ions and •OH generated by Fenton−like reactions can easily enter the interior of the bacteria to induce damage. It is believed that metal−doped PDA coating can solve the key problem of enhancing the effective ROS production capacity of CDT and provide guidance for the design of multifunctional antibacterial materials.

## Data Availability

Data available on request due to restrictions eg privacy or ethical.
